# Tubulin Beta-3 Chain as a New Candidate Protein Biomarker of Human Skin Aging: A Preliminary Study

**DOI:** 10.1155/2017/5140360

**Published:** 2017-05-23

**Authors:** Sylvia G. Lehmann, Sandrine Bourgoin-Voillard, Michel Seve, Walid Rachidi

**Affiliations:** ^1^University Grenoble Alpes, ISTerre, CS 40700, 38058 Grenoble Cedex 9, France; ^2^University Grenoble Alpes, LBFA et BEeSy, PROMETHEE Proteomic Platform, Grenoble, France; ^3^Inserm, U1055, PROMETHEE Proteomic Platform, Grenoble, France; ^4^CHU Grenoble Alpes, Institut de Biologie et de Pathologie, PROMETHEE Proteomic Platform, Grenoble, France; ^5^University Grenoble Alpes, INAC, SyMMES, 38000 Grenoble, France; ^6^CEA, INAC, SyMMES, 38054 Grenoble, France

## Abstract

Skin aging is a complex process, and a lot of efforts have been made to identify new and specific targets that could help to diagnose, prevent, and treat skin aging. Several studies concerning skin aging have analyzed the changes in gene expression, and very few investigations have been performed at the protein level. Moreover, none of these proteomic studies has used a global quantitative labeled proteomic offgel approach that allows a more accurate description of aging phenotype. We applied such an approach on human primary keratinocytes obtained from sun-nonexposed skin biopsies of young and elderly women. A total of 517 unique proteins were identified, and 58 proteins were significantly differentially expressed with 40 that were downregulated and 18 upregulated with aging. Gene ontology and pathway analysis performed on these 58 putative biomarkers of skin aging evidenced that these dysregulated proteins were mostly involved in metabolism and cellular processes such as cell cycle and signaling pathways. Change of expression of tubulin beta-3 chain was confirmed by western blot on samples originated from several donors. Thus, this study suggested the tubulin beta-3 chain has a promising biomarker in skin aging.

## 1. Introduction

Life expectancy in developed countries over the past two centuries has considerably increased, and if this trend continues through the 21st century, most babies born since 2000 in such countries will reach 100 years. Also, it is expected that by 2030, one in eight people worldwide will be 65 or above and the global aging of the population will lead to several societal, economical, and medical challenges [1].

Aging is a complex process influenced by multiple genetic and environmental factors and is characterized by a progressive decline in multiple physiological functions. Skin like other organs is affected by aging that can be accelerated by environmental factors such as UV radiation. Intrinsic skin aging is observed in sun-nonexposed skin and reflects the aging process of the entire organism [2]. Thereby, skin is an interesting alternative approach to decipher the intrinsic aging process as it is easily accessible compared to internal organs or tissues. Skin undergoes several morphological and physiological changes with intrinsic aging such as fine wrinkle formation, thinning of the epidermis and dermis, increased vulnerability and fragility, dryness, loss of elasticity, and disturbed barrier function [2]. The underlying mechanisms of intrinsic aging are multiple: cellular senescence and decreased proliferative capacity; shortening of the telomeres; increase in DNA damage and reduction in DNA repair processes; mitochondrial and genomic DNA mutations; hormonal decline and oxidative stress [3, 4].

Over the last decade, several transcriptomic studies have investigated the effect of aging on gene expression in several organism models and in humans [5, 6]. Concerning skin aging, only few studies have been conducted in humans. The first showed that genes differently expressed in elderly and young human male skin were involved in various cellular processes such as metabolism, signal transduction, apoptosis, and regulation of transcription [7]. More recently, a study has compared the gene expression profile from sun-nonexposed skin in both genders depending on aging. There was a significant different response in both genders with aging, with only 39 genes commonly dysregulated and 4 of them regulated in the opposite manner in both genders. From these results, the WNT signaling pathway has emerged as the major downregulated pathway with aging in both sexes [8]. And lately, 75 differentially expressed genes were identified in human epidermis according to age status [9]. Pathway analysis revealed that these genes were mainly involved in cell migration, cancer, dermatological diseases, and cell proliferation. Also, genes involved in the development of the epidermis were significantly enriched, and an overall downregulation of keratinocytes differentiation was observed.

Proteins are the workhorses of the cell and the main effectors of numerous cellular processes. Quantitative mass spectrometry-based proteomics has proven its utility for the description of protein dynamics in order to decipher complex processes and to describe normal and pathological states [10–13]. Relatively few studies have used proteomic to investigate skin aging, and they all used a two-dimensional gel electrophoresis approach which leads to a lower coverage of the proteome than gel-free approaches that could provide a proteomic signature of aging [14–16].

Skin is composed of several cell layers, and previous studies have investigated the protein expression level in whole skin or in *stratum corneum* samples. But the different cell layers can behave very differently and exhibit a different response to aging. Keratinocytes are the major components of the epidermis which is the most superficial and accessible layer of the skin. Focusing on keratinocytes will help to gain a deeper insight into skin aging and to have access to the proliferative compartment of the epidermis. Skin structure differs between genders, mainly due to the role of androgens in skin morphology. In humans, male skin is thicker than female skin and female subcutaneous tissues are thicker than male's one [17]. We then investigated the changes in the protein expression profile in human primary keratinocytes derived from sun-nonexposed skin obtained from young and elderly Caucasian women (mean age 62.5 years, *n* = 2, and 29.5 years, *n* = 2, resp.). Considering that the skin undergoes significant changes during menopause [18], the age categories were chosen so that the elderly women would have more probably reached menopause. Indeed, the average age where women reached menopause was 48.8 ± 4 years in a French cohort of women [19].

Our quantitative proteomic profiling, which expression was significantly dysregulated, of young and elderly primary human keratinocytes identified 58 proteins that are putative candidate biomarkers for intrinsic skin aging. Further western blot analysis on 14 donors confirms that tubulin beta-3 chain could be a biomarker of skin aging.

## 2. Material and Methods

### 2.1. Cell Culture

Human keratinocyte cultures were established by outgrowth from skin biopsies obtained after plastic mammary surgery (Centre Hospitalier Universitaire Grenoble Alpes, France) from healthy donors with their informed consent. Donors were Caucasian women aged 57–71 years (*n* = 10) and 18–32 years (*n* = 8) classified in two age groups designed hereafter elderly and young, respectively. Skin biopsies were from sun-nonexposed skin. Isolation and culture of primary keratinocytes in KSFM medium supplemented with 25 *μ*g/mL bovine pituitary extract (BPE), 1.5 ng/mL EGF (Life Technologies), and 75 *μ*g/mL Primocin™ (InvivoGen) were done as previously described [20]. To avoid replicative senescence, cells were used at passage 2 or 3.

### 2.2. Protein Extraction for MS Analysis

Cultured keratinocytes obtained from young (27 and 32 years) and elderly (60 and 65 years) donors were cultivated up to passage 2 and harvested by trypsinization. After two washes with PBS (Life Technologies), cell pellets were kept at −80°C up to the extraction. Frozen cell pellets were lysed for 30 minutes at 4°C in a solution containing 40 mM HEPES pH 7.4, 100 mM NaCl, 1 mM EDTA, 0.02% Triton, 0.02% sodium deoxycholate, 0.2 mM TCEP, and protease and phosphatase inhibitor cocktails (PhosSTOP) from Roche. Lysis was achieved by short sonication on ice, and the lysates were cleared by centrifugation at 14,000 rpm for 20 minutes at 4°C. The concentration of the protein extract was determined using BCA protein assay kit (Thermo Fisher Scientific, IL, USA).

### 2.3. Protein Digestion and iTRAQ Labeling

Protein samples were labeled with iTRAQ reagents according to the manufacturer's instructions (iTRAQ Reagents 8 plex Applications kit; Sciex, Framingham, MA, USA). Briefly, equal amount of protein extract obtained from cells originated from young donors was pooled in order to achieve a total of 100 *μ*g of proteins. The same procedure was applied for cells from elderly donors. The samples were reduced in 20 mM of TCEP (tris-(2-carboxyethyl)phosphine) at 37°C for 1 h, and cysteine residues were blocked in 10 mM of MMTS (methyl methanethiosulfonate) at room temperature for 10 min, followed by trypsin (Promega, Lyon, France) digestion at a ratio of 1 : 10 (trypsin : protein) at 37°C overnight. Each peptide solution was labeled with one iTRAQ reagent: iTRAQ reporter ions of *m/z* 113.1 for young and *m/z* 117.1 for elderly. iTRAQ labeling was verified for all reactions, and the samples were pooled in a ratio 1 : 1 and dried by vacuum centrifugation prior to the OFFGEL peptide fractionation.

### 2.4. Peptide OFFGEL Isoelectrofocusing

Peptide fractionation according to their pI was performed with 3100 OFFGEL Fractionator and the OFFGEL Kit linear pH 3–10 (Agilent Technology, Les Ulis, France) in a 24-well setup following the manufacturer's instructions. The device was set up for the 24-fraction separation by using a 24-cm-long IPG gel strip with a linear pH gradient ranging at 3–10. iTRAQ-labeled peptide mix was dried by vacuum centrifugation and resuspended in focusing OFFGEL buffer prior to loading in each of the 24 wells. Peptides were focused with a constant current of 50 *μ*A until 50 kVh was reached. After complete fractionation, peptide samples were recovered from each well, dried in a vacuum concentrator, and then desalted using C18 ZipTips (Millipore, MA, USA).

### 2.5. Reversed Phase Nanoliquid Chromatography

Further peptide separation was performed on an Ultimate 3000 C18 reversed phase nanoliquid chromatography (RP-nanoLC) system (Ultimate 3000, Dionex/Thermo Scientific) controlled by Chromeleon v. 6.80 software (Dionex/Thermo Scientific/LC Packings, Amsterdam, The Netherlands) and coupled with a PROBOT MALDI spotting device controlled by the *μ*Carrier 2.0 software (Dionex/Thermo Scientific/LC Packings, Amsterdam, The Netherlands). Vacuum dried fractions were resuspended in buffer A (98% water, 2% ACN, and 0.05% TFA) before injection on a nanotrapping column (C18, 3 *μ*m, 100 Å pore size; LC Packing) in 2% ACN and 0.05% TFA at a flow rate of 20 *μ*L/min for 5 min. Then, trapped peptides were separated by reversed phase chromatography (*Acclaim PepMap300* 75 *μ*m, 15 cm, nanoViper C18, 3 *μ*m, 100 Å pore size; Thermo Scientific) with a binary gradient of buffer A (2% ACN and 0.05% TFA) and buffer B (80% ACN and 0.04% TFA) at a flow rate of 0.3 *μ*L/min. The entire run lasted 60 min, and the nanoLC gradient was set up as follows: 5–35 min, 8–42% B; 35–40 min, 42–58% B; 40–50 min, 58–90% B; and 50–60 min, 90% B. Fractions from eluted solution were collected and spotted on a MALDI sample plate (Sciex, Les Ulis, France) at a frequency of one spot per 15 seconds. The *α*-cyano-4-hydroxy-cinnamic acid matrix (HCCA, 2 mg/mL in 70% ACN and 0.1% TFA) was continuously added to the column effluent at a flow rate of 0.9 *μ*L/min and, therefore, integrated in each spot of MALDI sample plate.

### 2.6. MALDI-TOF/TOF Analysis

MS and MS/MS analysis of nanoLC-off-line spotted peptide samples was performed using the 4800 MALDI-TOF/TOF mass spectrometer (Sciex, Les Ulis, France) controlled by the 4000 Series Explorer software v. 3.5. The mass spectrometer was operated in a positive reflector mode. Each spectrum was externally calibrated using the Peptide Calibration Standard II (Bruker Daltonics, Bremen, Germany), and the peptide mass tolerance was set to 50 ppm. MS spectra were acquired in a *m/z* 700–4000 range. Up to 30 of the most intense ions per spot position characterized by a *S*/*N* (signal/noise) ratio higher than 40 were chosen for MS/MS analysis. Selected ions were activated by using CID (collision-induced dissociation) in order to obtain the corresponding MS/MS spectrum.

### 2.7. Analysis of iTRAQ Data

MS and MS/MS spectra were used for identification and relative quantitation by using ProteinPilot™ software v 4.0 with the Paragon™ (Sciex, Les Ulis, France) and Mascot (Matrix Science, London, UK) search engines. The analysis was performed with the UniProtKB database released on June 2015, and the taxonomy was limited to *Homo sapiens*. Concerning Paragon search engine, the search effort was set to “Thorough ID” and the False Discovery Rate Analysis (FDR) of 1% was applied. For quantification, bias and background correction was applied and only quantified proteins with at least 1 peptide at the 95% peptide confidence level were included. For Mascot search engine, the FDR was set lower than 1% and only peptides with a score higher than 30 were considered. Data were merged at the peptide level after Paragon and Mascot analysis. In order to obtain a high-quality quantitative analysis, we analyzed our data with the R package Isobar [21] which allows the determination of statistical significance of protein/peptide regulation. A normal fit was used and only proteins which ratio had a *p* value ratio and a *p* value sample <0.05 are then considered as significantly differently expressed depending on age. For output of our quantitative iTRAQ results, all protein ratios were expressed as elderly over young (117 : 113) to present relative protein quantification ratios. A summary of the parameters applied for the mass data analysis is presented in Supplemental Data (Table S1) available online at https://doi.org/10.1155/2017/5140360.

### 2.8. Gene Ontology and Pathway Analysis

Gene ontology and protein function analysis were performed using PANTHER (http://www.pantherdb.org/) [22] by importing the list of dysregulated proteins. Each protein was classified into one or several categories regarding PANTHER family, protein class, GO-slim molecular function, biological process, cellular component, and pathway. This functional classification of proteins regarding biological process and protein class is graphically illustrated in a pie chart. Pathway enrichment analysis was performed with PathVisio 3.2.2 Revision: 4047 [23] by importing the list presented in Table S2. The following criteria have been used [Log10 ratio] < −0.15 OR [Log10 ratio] > 0.16 AND [is significant] = 1 and the calculation method was pathway-centric. The top enriched pathways and their *Z*-scores are represented.

### 2.9. Western Blot Analysis

Human primary keratinocytes were harvested and cultivated as described previously from skin biopsies of 8 young (age: 18, 21, 24, 26, 27 (2 donors), 30, and 32) and 10 elderly donors (age: 57, 59, 60, 62 (2), 65 (2), 66, 68, and 71). At early passages (2 or 3, when cells are still proliferating), cells were lysed by vortexing in RIPA Buffer (Sigma-Aldrich) containing protease inhibitors (Complete Mini protease inhibitor cocktail, Roche, Switzerland), 1 mM DTT and 100 *μ*M PMSF. Samples were then centrifuged for 15 minutes at 14,000 rpm and the supernatants collected. Protein concentration was determined with MicroBC Assay (Interchim), and 20 *μ*g of total protein was loaded on TGX Stain-Free™ FastCast™ 12% Acrylamide gels (Biorad). Proteins were transferred onto a nitrocellulose membrane using Trans-Blot® Turbo™ Transfert System (Biorad). Membranes were blocked with TBS-Tween 0.5% containing 5% nonfat milk and incubated with the tubulin beta-3 chain antibody (MA1-118; Thermoscientific) at 1/1000 dilution in TBS-Tween 0.5% containing 5% nonfat milk overnight at 4°C. After washing in TBS-Tween 0.5%, membranes were incubated with HRP conjugated secondary antibodies (Amersham ECL anti-mouse IgG HRP-linked, whole antibody, GE Healthcare) for 1 h at RT. Membranes were then washed in TBS-Tween 0.5%, and blot images were acquired on Molecular Imager Gel Doc XR+ and Chemidoc XRS+ Systems (Biorad). Specific detected bands were quantified with Image LAb 2.0 Software (Biorad), and corresponding intensities were normalized with total protein content and expressed as a ratio.

Western blot results of tubulin beta-3 chain were illustrated by box plots, and receiver operating characteristic curve (ROC curve) was created by using GraphPad Prism version 7.00 for Windows (GraphPad Software, La Jolla California USA, http://www.graphpad.com).

## 3. Results

### 3.1. Identification of Fifty-Eight Proteins Differentially Expressed with Aging by Proteomic Analysis

In order to obtain a quantitative proteomic map of elderly and young donor-derived keratinocyte cells, we used an iTRAQ labeling coupled with OFFGEL fractionation and off-line nanoLC/MS/MS as previously described [24]. The bioinformatics analysis with Paragon and Mascot search engines resulted in the identification of 517 unique proteins using a 1% FDR and considering only proteins with at least 1 peptide with confidence level ≥ 95% and score > 30. We performed a statistical analysis with the isobar package and quantified 446 proteins. Elderly keratinocytes were labeled with iTRAQ *m/z* 117 tag and young keratinocytes with iTRAQ *m/z* 113 tag. Thus, the ratio 117 : 113 (Elderly : Young) indicates the relative protein abundance between elderly and young cell samples. The complete list of identified proteins, including the UniProtKB accession number, ID, protein and gene name, peptide count, spectral count, sequence coverage, iTRAQ ratios with corresponding *p* value ratio and *p* value sample for elderly versus young cells are provided in Supplemental Data (Table S2). When the *p* value ratio and the *p* value sample were both <0.05, proteins were considered significantly differently expressed. Applying these criteria, we identified 58 proteins significantly differentially expressed depending on age status. From them, 40 were downregulated and 18 were upregulated with aging (Tables 1 and 2).

### 3.2. Gene Ontology Analysis

**Figure 1:**
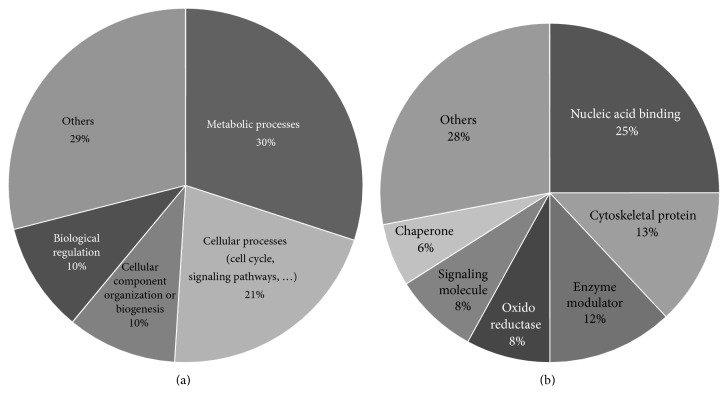
Functional distribution of the 58 proteins identified as dysregulated with aging according to biological processes (a) and PANTHER protein class (b) categories. Assignments were made with PANTHER tool. The numbers in brackets correspond to the percentage of identified proteins classified in the category. If a protein is classified into 2 ontology terms that are not parent or child to each other, it counts in the 2 classes.

The 58 proteins previously identified were analyzed using PANTHER [22]. Their classifications into gene ontology and PANTHER categories are as follows: protein family, protein class, molecular function, biological process, cellular component, and pathway is listed in Table 3. This functional classification of biological process and PANTHER protein class is graphically illustrated in Figure 1(). The main represented biological process categories are metabolism (30%); cellular process including cell cycle/cell signaling pathways/cell component movement (21%); cellular component organization/biological regulation (10%); localization/developmental process (8%); response to stimuli (4%); multicellular organismal process/immune system process (3%); and biological adhesion (1%). Concerning protein class, dysregulated proteins belong to the main following protein classes: nucleic acid binding (25%); cytoskeletal protein (13%); enzyme modulator (12%); oxidoreductase and signaling molecules (8%); chaperone (6%); transferase/transcription factor (4%); and extracellular matrix protein/hydrolase/carrier protein/membrane traffic protein/cell junction protein/kinase/isomerase/receptor (2%).

Pathway enrichment analysis was performed using PathVisio [23], and the top enriched pathways are listed in Table 4. Metal homeostasis is in the top enriched pathways, as well as histone modifications, DNA replication, oxidative stress, and electron transport chain pathways.

### 3.3. Western Blot Analysis of Tubulin Beta-3 Chain, a Promising Candidate Protein Biomarker of Aging

**Figure 2:**
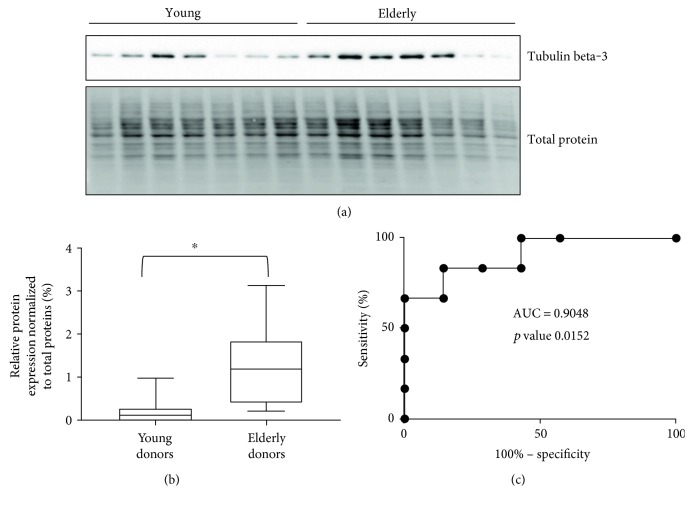
(a) Western blot analysis of protein extract from young and elderly keratinocytes with tubulin beta-3 chain. (b) Box plots of relative protein expression of tubulin beta-3 chain in young and elderly donors (based on western blot data by considering the relative intensity of the specific antibodies on the membrane versus the amount of protein loaded on the gel for young and elderly donors, ^∗^significant *p* value < 0.05 (*p* value 0.0152)). (c) Receiver operating characteristic curve (ROC curve) with an AUC of 0.9048.

In proteomic quantitation analysis, tubulin beta-3 chain was evidenced as a promising candidate protein biomarker of aging. Indeed, tubulin beta-3 chain was upregulated in elderly donors with an iTRAQ ratio of protein expression level of 2.66 (elderly versus young) and significant *p* values (*p* value ratio of 4.21 × 10^−4^ and a *p* value sample of 4.87 × 10^−7^). Thus, tubulin beta-3 chain was further analyzed by western blot on human primary keratinocyte cells from the same donors but also from at least fourteen other donors in order to exclude the interindividual variability and to figure out whether or not these proteins were dysregulated in other donors. For this western blot analysis, total protein quantification was used as a control of gel loading and relative intensity of the specific antibodies on the membrane versus the amount of protein loaded on the gel was calculated and represented on box-plot diagram. Representative western blot images and results of quantification are shown in Figure 2(). The trend of dysregulation of tubulin beta-3 chain was confirmed, and a similar ratio was obtained by western blot quantification and with our proteomic workflow. Moreover, receiver operating characteristic curve (ROC curve) confirmed the specificity and the sensitivity of tubulin beta-3 chain expression level for determination of age status with an area under the ROC curve of 0.9048 and a *p* value of 0.0152.

## 4. Discussion

Skin aging is a complex process with multifactorial origins that can decipher using new technological approach such as global quantitative proteomics. We carried out an iTRAQ-MALDI-TOF/TOF MS and MS/MS analysis to identify and quantify changes in human primary keratinocyte proteomes from young and elderly donors. 517 proteins were identified including proteins found mainly in keratinocytes such as cornifin-B and keratin-2E which are associated with keratinocyte activation, proliferation, and keratinization [25]. After applying robust statistical analysis, 58 proteins were found significantly differentially expressed depending on age status with 40 that were downregulated and 18 upregulated with aging.

Comparison of our results with previous gene and protein expression studies of skin aging shows some similarities. We found that more proteins are downregulated (40) than upregulated (18) with aging which is consistent with the previous results from a gene expression study in women [8]. The majority of proteins which expression is affected by age are nucleic acid-binding proteins (25%), cytoskeletal proteins (13%), and enzyme modulators (12%). These proteins are mostly involved in metabolism (30%) or cellular process (21%) such as cell cycle and cell signaling pathways. Similar results have been observed in a previous transcriptomic study [7]. Pathway enrichment analysis reveals metal (zinc and copper) homeostasis as one of the most enriched pathways which is consistent with the observed dysregulation of several metallothionein proteins that bind diverse types of metal and also participated in oxidative stress response [26]. Oxidative stress and electron transport chain pathways are also in the enriched pathways' list, and this is consistent with the increase of reactive oxygen species (ROS) production with aging [3].

In this work, tubulin beta-3 chain expression is upregulated with aging in our proteomic experiment and also in western blot analyses on samples from several donors. Statistical analysis of these data has shown that tubulin beta-3 chain may discriminate age status. Tubulin beta-3 chain is a component of the microtubules that are complex polymers composed of tandem repeats of *α*- and *β*-tubulin heterodimers. In humans, six *β*-tubulin isoforms have been described and the isoform composition of the microtubule is determining its behavior. For example, it has been shown that in vitro purified microtubules enriched in tubulin beta-3 chain are more dynamic compared to microtubules containing other *β*-tubulin isoforms [27]. Tubulin beta-3 chain mutations have been linked in humans to different types of neurological disorders with abnormal axon guidance [28, 29]. Tubulin beta-3 was also reported as a biomarker for melanocyte lineage and as involved in melanocyte differentiation and melanogenesis [30], and it has been shown that tubulin beta-3 expression is decreased in senescent melanocytes [31]. Locher et al. [30] also reported that tubulin beta-3 may play a substantial role in melanosome transport which is regulated by proteins (such as kinesin and dynein) using microtubule tracks. Moreover, melanosomes may be transferred from melanocytes to keratinocytes through the shedding vesicle system [32], but the cellular process is still unclear and no data was reported concerning ageing aspects. Interestingly, tubulin beta-3 was identified as a direct downstream protein of human melanocortin 1 receptor (MC1R) [33], a protein associated to skin pigmentation, ultraviolet radiation, and to other aspects such as skin cancers [34]. High level of tubulin beta-3 chain has been linked to resistance to antitubulin agents such as taxanes and vinorelbine and lower overall survival in nonsmall cell lung cancer (NSCLC) [35–37] and prostate tumor patients [38]. But the prognostic value of tubulin beta-3 chain is still debated in cutaneous malignant melanoma prognosis [39]. The high expression of tubulin beta-3 chain in NSCLC has been shown to be regulated by ras, PI3/akt, and MAP kinase-ERK signaling [40]. Tubulin beta-3 chain has also been described to be a prognostic marker for bladder urothelial carcinoma with patients showing a higher level of tubulin beta-3 chain presenting a shorter disease-free survival [41]. To date, no publication reported tubulin beta-3 chain protein as a candidate biomarker for aging and there is no known mechanism of tubulin beta-3 chain dysregulation in skin aging. But it is interesting to notice that alterations of the cytoskeleton have been reported with aging [7] and that mutations in lamin genes (type V intermediate filaments) are responsible for some premature aging diseases such as the Hutchinson-Gilford progeria syndrome (HGPS) [42].

In our study, we identified other dysregulated proteins such as peroxiredoxin 3, 6-phosphofructokinase, platelet type, and cornifin-B. Thioredoxin-dependent peroxide reductase, mitochondrial, also known as peroxiredoxin 3 (PrxIII), a mitochondrial member of the antioxidant family of thioredoxin (Trx) peroxidases, was found upregulated with aging in our study. Two other family members, peroxiredoxins 1 and 2, were also upregulated in a previous report [14]. Peroxiredoxins are important cellular antioxidant; indeed, they act as hydrogen peroxide and organic hydroperoxide scavengers [43]. It is well established that with age, there is an increase in reactive oxygen species (ROS) production and a decrease in antioxidant activity both contributing to chronological aging [3]. In oxidative stress conditions, PrxIII undergoes overoxidation and subsequent irreversible inactivation. And it has been shown that in rats, this modified PrxIII form accumulates with aging [44]. In our analysis, we did not identify the peptide containing the cysteine that is overoxidized to sulfonic acid and we then cannot discriminate between the two forms, explaining why, in consequence, we observed a global upregulation of the protein. Concerning 6-phosphofructokinase, platelet type (ATP-PFK) which is upregulated with aging in our study, this enzyme catalyzes the phosphorylation of D-fructose 6-phosphate to fructose 1,6-bisphosphate by ATP, the first committing step of glycolysis. It has been shown that human primary keratinocytes derived from elderly donors showed higher glucose uptake and increased lactate production, which are the indicators of a shift in metabolism towards increased glycolysis [45]. Thus, the observed upregulation of ATP-PFK is correlated with the increased glycolysis in primary keratinocytes. Interestingly, cornifin-B is a marker of keratinocyte differentiation [46] and two transcriptomic studies reported that cornifin-B was downregulated with aging using women epidermis [9] and skin biopsies [47]. Our study is in concordance with this view as our proteomic study showed a downregulation of cornifin-B expression with aging (iTRAQ ratio erlderly versus young of 0.405 with a *p* value ratio of 2.54 × 10^−2^ and a *p* value sample of 3.18 × 10^−6^).

Comparing our results with other studies aimed at identifying biomarkers of skin aging shows some differences that may be explained by different type/origin of skin samples, gender, difference in sample processing all along the workflow, and the variable correlation between mRNA and protein expression levels [48]. But using different and complementary approaches is interesting as it can lead to the identification of even more candidate biomarkers than with a single method. Also, considering the occurrence of oxidative stress during aging and the subsequent induced altered posttranslational modifications (PTMs) of proteins such as carbonylation, 3-nitrityrosilation, emphasis must be placed on the study of PTMs with aging in future proteomic experiment concerning skin aging [49].

## 5. Conclusions

Defining the differential protein signature with aging even if these changes could be initiating adaptive or compensatory events is crucial to further increase our knowledge of skin aging. Our aim was to identify some biomarker candidates for skin aging from human primary keratinocyte culture. 58 proteins were dysregulated with aging, and from this, tubulin beta-3 chain was also observed dysregulated in western blot analysis of keratinocyte extracts isolated from multiple donors. Statistical analysis has confirmed especially that an increase in tubulin beta-3 chain is associated with aging. Further studies will be needed in order to evaluate the effect of this change of expression on the complex process of aging.

This study brings a new effort to reach a better understanding of the biology of skin aging and to identify new and specific targets that could help to diagnose, prevent, and treat skin aging. Indeed, emerging diagnostic tools now require a combination of multiple biomarkers to achieve a better accuracy, and we propose that tubulin beta-3 chain could be one of these.

## Supplementary Material

Supplemental Table 1: Summary of the parameters applied for the bioinformatic analysis and results obtained. Supplemental Table 2. Protein identification (A and B) and relative quantitation (A) summary generated with isobar after combining Mascot and Protein Pilot reports from iTRAQ-based OFFGEL-LC-MALDI TOF/TOF analyses. The file contains 517 proteins that were identified and 446 proteins quantified, with the Accession / variants, Protein ID, Description, Gene name, Peptide Count (number of specific peptides), Spectral Count (number of specific spectra), Sequence Coverage and where applicable the Ratio [Elderly / Young], Log10 Ratio, and statistical values such as is Significant, P Value Ratio and P Value Sample. Protein expression level in elderly cells are normalized to protein expression level in young cells (iTRAQ ratio 117/113).

## Figures and Tables

**Table 1:** List of proteins significantly downregulated in elderly versus young cells (iTRAQ ratio 117/113). Statistically significant iTRAQ ratios (*p* value ratio and *p* value sample ≤ 0.05) for the 40 proteins that are downregulated.

Accession/variants	ID	Description	Gene	Peptide count	Spectral count	Sequence coverage (%)	Ratio [elderly/young]	*p* value rat	*p* value sample	Log10 ratio
O60814	H2B1K_HUMAN	Histone H2B type 1-K	HIST1H2BK	9	131	4.76	0.170	6.27*E*−05	4.36*E*−19	−0.769
O75334-[2-6]	LIPA2_HUMAN	Liprin-alpha-2	PPFIA2	2	2	0.64	0.295	3.83*E*−02	5.16*E*−10	−0.531
P20674	COX5A_HUMAN	Cytochrome c oxidase subunit 5A, mitochondrial	COX5A	2	3	9.33	0.309	1.56*E*−02	2.23*E*−09	−0.510
P04732	MT1E_HUMAN	Metallothionein-1E	MT1E	1	9	16.39	0.310	7.22*E*−13	2.40*E*−09	−0.509
P05204	HMGN2_HUMAN	Nonhistone chromosomal protein HMG-17	HMGN2	4	11	8.89	0.330	1.89*E*−02	1.50*E*−08	−0.482
Q15075	EEA1_HUMAN	Early endosome antigen 1	EEA1	2	3	0.64	0.331	1.71*E*−02	1.61*E*−08	−0.481
Q9UKY7-[2]	CDV3_HUMAN	Protein CDV3 homolog	CDV3	3	16	11.63	0.342	1.18*E*−03	4.11*E*−08	−0.466
O75152	ZC11A_HUMAN	Zinc finger CCCH domain-containing protein 11A	ZC3H11A	1	1	1.48	0.349	2.87*E*−02	7.18*E*−08	−0.457
P17096	HMGA1_HUMAN	High mobility group protein HMG-I/HMG-Y	HMGA1	1	19	7.48	0.352	1.56*E*−02	8.83*E*−08	−0.454
P06454-[2]	PTMA_HUMAN	Prothymosin alpha [cleaved into: prothymosin alpha, N-terminally processed; thymosin alpha-1]	PTMA	5	24	12.61	0.357	4.01*E*−02	1.34*E*−07	−0.447
Q8NC51-[3]	PAIRB_HUMAN	Plasminogen activator inhibitor 1 RNA-binding protein	SERBP1	11	90	4.66	0.361	1.56*E*−02	1.82*E*−07	−0.442
Q13442	HAP28_HUMAN	28 kDa heat- and acid-stable phosphoprotein	PDAP1	1	1	7.18	0.363	1.23*E*−02	2.09*E*−07	−0.440
P63313	TYB10_HUMAN	Thymosin beta-10	TMSB10	2	35	13.64	0.364	1.30*E*−02	2.21*E*−07	−0.439
O00233	PSMD9_HUMAN	26S proteasome non-ATPase regulatory subunit 9	PSMD9	1	6	5.38	0.383	2.34*E*−04	8.33*E*−07	−0.416
P05114	HMGN1_HUMAN	Nonhistone chromosomal protein HMG-14	HMGN1	3	13	8.00	0.384	1.20*E*−02	8.80*E*−07	−0.415
P62158	CALM_HUMAN	Calmodulin	CALM1	10	147	8.72	0.385	2.89*E*−02	9.13*E*−07	−0.415
P02795, P13640-[2], P80297	MT1G_HUMAN, MT1X_HUMAN, MT2_HUMAN	Metallothionein-1G, Metallothionein-1X, Metallothionein-2	MT1G, MT1X, MT2A	1	20	16.29	0.392	3.61*E*−03	1.47*E*−06	−0.406
P67936	TPM4_HUMAN	Tropomyosin alpha-4 chain	TPM4	5	48	3.23	0.401	3.64*E*−02	2.51*E*−06	−0.397
P22528	SPR1B_HUMAN	Cornifin-B	SPRR1B	4	56	8.99	0.405	2.54*E*−02	3.18*E*−06	−0.392
Q92538-[2,3]	GBF1_HUMAN	Golgi-specific brefeldin A-resistance guanine nucleotide exchange factor 1	GBF1	1	1	0.32	0.406	4.48*E*−02	3.34*E*−06	−0.391
P51858	HDGF_HUMAN	Hepatoma-derived growth factor	HDGF	4	12	3.75	0.429	2.37*E*−02	1.17*E*−05	−0.368
P61604	CH10_HUMAN	10 kDa heat shock protein, mitochondrial	HSPE1	12	121	7.84	0.455	2.20*E*−02	4.16*E*−05	−0.342
P07108-[2-5]	ACBP_HUMAN	Acyl-CoA-binding protein	DBI	4	50	9.20	0.460	1.86*E*−02	5.21*E*−05	−0.337
Q9C030-[2]	TRIM6_HUMAN	Tripartite motif-containing protein 6	TRIM6	2	8	1.23	0.463	1.10*E*−02	6.03*E*−05	−0.334
P20962	PTMS_HUMAN	Parathymosin	PTMS	4	11	8.82	0.468	1.44*E*−02	7.30*E*−05	−0.330
Q9GZP8	IMUP_HUMAN	Immortalization upregulated protein	IMUP	3	5	9.43	0.476	8.05*E*−03	1.05*E*−04	−0.322
Q9H299	SH3L3_HUMAN	SH3 domain-binding glutamic acid-rich-like protein 3	SH3BGRL3	3	36	10.75	0.490	1.62*E*−02	1.82*E*−04	−0.310
P62857	RS28_HUMAN	40S ribosomal protein S28	RPS28	3	20	17.39	0.491	9.21*E*−03	1.93*E*−04	−0.309
P16949-[2]	STMN1_HUMAN	Stathmin	STMN1	3	24	8.72	0.492	2.51*E*−02	2.00*E*−04	−0.308
P02765	FETUA_HUMAN	Alpha-2-HS-glycoprotein	AHSG	4	61	3.27	0.507	4.77*E*−02	3.38*E*−04	−0.295
O15212	PFD6_HUMAN	Prefoldin subunit 6	PFDN6	1	9	9.30	0.519	1.72*E*−02	5.20*E*−04	−0.285
P52926	HMGA2_HUMAN	High mobility group protein HMGI-C	HMGA2	3	8	11.93	0.528	1.02*E*−04	7.07*E*−04	−0.277
P61956	SUMO2_HUMAN	Small ubiquitin-related modifier 2	SUMO2	1	7	12.63	0.534	1.89*E*−02	8.65*E*−04	−0.272
Q9UHV9	PFD2_HUMAN	Prefoldin subunit 2	PFDN2	1	5	9.09	0.544	1.81*E*−02	1.16*E*−03	−0.265
P20929-[2,3]	NEBU_HUMAN	Nebulin	NEB	4	4	0.13	0.563	5.23*E*−06	2.03*E*−03	−0.250
P09429	HMGB1_HUMAN	High mobility group protein B1	HMGB1	5	33	5.58	0.563	9.96*E*−07	2.07*E*−03	−0.249
P62328	TYB4_HUMAN	Thymosin beta-4	TMSB4X	1	16	15.91	0.582	4.60*E*−03	3.41*E*−03	−0.235
P09497-[2]	CLCB_HUMAN	Clathrin light chain B	CLTB	7	24	3.49	0.593	1.87*E*−03	4.46*E*−03	−0.227
P35749-[2-4]	MYH11_HUMAN	Myosin-11	MYH11	1	5	0.56	0.649	4.13*E*−02	1.53*E*−02	−0.188
Q16629-[2-4]	SRSF7_HUMAN	Serine/arginine-rich splicing factor 7	SRSF7	2	13	3.78	0.651	3.09*E*−02	1.59*E*−02	−0.187

**Table 2:** List of proteins significantly upregulated in elderly versus young cells (iTRAQ ratio 117/113). Statistically significant iTRAQ ratios (*p* value ratio and *p* value sample ≤ 0.05) for the 18 proteins that are upregulated.

Accession/variants	ID	Description	Gene	Peptide count	Spectral count	Sequence coverage (%)	Ratio [elderly/young]	*p* value ratio	*p* value sample	Log10 ratio
P26373	RL13_HUMAN	60S ribosomal protein L13	RPL13	4	15	4.27	1.455	2.54*E*−03	3.04*E*−02	0.163
P01861	IGHG4_HUMAN	Ig gamma-4 chain C region	IGHG4	1	4	4.89	1.482	2.00*E*−02	2.46*E*−02	0.171
Q13200	PSMD2_HUMAN	26S proteasome non-ATPase regulatory subunit 2	PSMD2	1	5	1.65	1.511	4.13*E*−02	1.96*E*−02	0.179
P13797	PLST_HUMAN	Plastin-3	PLS3	5	13	1.90	1.584	2.13*E*−02	1.08*E*−02	0.200
P49721	PSB2_HUMAN	Proteasome subunit beta type-2	PSMB2	1	22	5.47	1.635	1.82*E*−03	7.01*E*−03	0.214
P48643	TCPE_HUMAN	T-complex protein 1 subunit epsilon	CCT5	6	35	1.29	1.696	2.17*E*−03	4.15*E*−03	0.229
P11166	GTR1_HUMAN	Solute carrier family 2, facilitated glucose transporter member 1	SLC2A1	4	46	2.03	1.891	2.60*E*−02	7.28*E*−04	0.277
P61158	ARP3_HUMAN	Actin-related protein 3	ACTR3	6	26	2.63	2.040	3.21*E*−02	1.84*E*−04	0.310
P09211	GSTP1_HUMAN	Glutathione S-transferase P	GSTP1	10	117	7.62	2.308	3.65*E*−02	1.47*E*−05	0.363
P78417	GSTO1_HUMAN	Glutathione S-transferase omega-1	GSTO1	4	25	5.81	2.482	2.89*E*−10	2.77*E*−06	0.395
P13667	PDIA4_HUMAN	Protein disulfide-isomerase A4	PDIA4	3	4	1.09	2.633	2.99*E*−02	6.60*E*−07	0.420
P62277	RS13_HUMAN	40S ribosomal protein S13	RPS13	1	1	7.95	2.638	2.88*E*−02	6.27*E*−07	0.421
Q13509	TBB3_HUMAN	Tubulin beta-3 chain	TUBB3	4	19	4.00	2.664	4.21*E*−04	4.87*E*−07	0.426
P30048	PRDX3_HUMAN	Thioredoxin-dependent peroxide reductase, mitochondrial	PRDX3	1	1	5.47	2.769	3.86*E*−02	1.80*E*−07	0.442
Q01813-[2]	PFKAP_HUMAN	ATP-dependent 6-phosphofructokinase, platelet type	PFKP	2	3	2.68	3.116	3.23*E*−02	6.75*E*−09	0.494
P50213	IDH3A_HUMAN	Isocitrate dehydrogenase [NAD] subunit alpha, mitochondrial	IDH3A	3	3	2.19	3.239	6.67*E*−03	2.14*E*−09	0.510
Q9Y6N5	SQRD_HUMAN	Sulfide:quinone oxidoreductase, mitochondrial	SQRDL	1	2	2.22	3.487	8.97*E*−03	2.17*E*−10	0.543
P38606-[2]	VATA_HUMAN	V-type proton ATPase catalytic subunit A	ATP6V1A	2	2	2.43	4.833	1.38*E*−02	1.75*E*−15	0.684

**Table 3:** List of proteins identified as differently expressed with age status and their functional classification relating to protein family, protein class, molecular function, biological process, cellular component, and pathway according to PANTHER and the GO database.

Accession	ID	Description	Gene	PANTHER family/subfamily	PANTHER protein class	PANTHER GO-slim molecular function	PANTHER GO-slim biological process	PANTHER GO-slim cellular component	Pathway
P61158	ARP3_ HUMAN	Actin-related protein 3	ACTR3	Actin-related protein 3 (PTHR11937:SF175)	Actin and actin-related protein(PC00085)	Structural constituent of cytoskeleton (GO:0005198)	Cytokinesis(GO:0009987); mitosis(GO:0000910); cellular component morphogenesis(GO:0007049); intracellular protein transport(GO:0007067); exocytosis(GO:0032502); endocytosis(GO:0009653); cellular component organization(GO:0032989)	Actin cytoskeleton(GO:0043226); intracellular(GO:0005856)	

P02765	FETUA_ HUMAN	Alpha-2-HS-glycoprotein	AHSG	Alpha-2-HS-glycoprotein (PTHR13814:SF6)	Extracellular matrix glycoprotein(PC00102); cysteine protease inhibitor(PC00100)	Cysteine-type peptidase activity(GO:0003824); protein binding (GO:0016787); cysteine-type endopeptidase inhibitor activity (GO:0008233)	Immune system process(GO:0002376); proteolysis(GO:0008152); mesoderm development(GO:0044238); skeletal system development(GO:0019538); regulation of catalytic activity (GO:0006508)	Extracellular region (GO:0005576); extracellular matrix (GO:0031012)	

P38606	VATA_ HUMAN	V-type proton ATPase catalytic subunit A	ATP6V1A	V-type proton atpase catalytic subunit A (PTHR15184:SF7)	ATP synthase(PC00227); anion channel (PC00068); ligand-gated ion channel (PC00002); ligand-gated ion channel (PC00133); DNA-binding protein (PC00049); hydrolase (PC00141)	Hydrolase activity (GO:0003824); receptor activity (GO:0016787); anion channel activity (GO:0004872); ligand-gated ion channel activity (GO:0005215); cation transmembrane transporter activity (GO:0022857); proton-transporting ATP synthase activity, rotational mechanism (GO:0005216); single-stranded DNA binding (GO:0005253)	Respiratory electron transport chain (GO:0008152); purine nucleobase metabolic process (GO:0006091); cation transport (GO:0022904)	Proton-transporting ATP synthase complex (GO:0032991); intracellular (GO:0043234)	

P62158	CALM_ HUMAN	Calmodulin	CALM1	Calmodulin (PTHR23050:SF216)	Calmodulin (PC00060)	Calcium ion binding (GO:0005488); calmodulin binding (GO:0005509)	Cellular component movement (GO:0009987); cell cycle (GO:0006928); cell communication (GO:0007049)		CCKR signaling map→CaM; B cell activation→calmodulin; heterotrimeric G-protein signaling pathway-rod outer segment phototransduction→ calmodulin; T cell activation→ calmodulin
P48643	TCPE_ HUMAN	T-complex protein 1 subunit epsilon	CCT5	T-complex protein 1 subunit epsilon (PTHR11353:SF94)	Chaperonin (PC00072)		Protein folding (GO:0008152); protein complex assembly (GO:0044238); protein complex biogenesis (GO:0019538)		

Q9UKY7	CDV3_ HUMAN	Protein CDV3 homolog	CDV3	Protein CDV3 homolog (PTHR16284:SF13)					

P09497	CLCB_ HUMAN	Clathrin light chain B	CLTB	Clathrin light chain B (PTHR10639:SF2)	Vesicle coat protein (PC00150)		Intracellular protein transport (GO:0051179); receptor-mediated endocytosis (GO:0006810)	Vesicle coat (GO:0032991); cytoplasm (GO:0043234)	Heterotrimeric G-protein signaling pathway-Gq alpha and Go alpha-mediated pathway→clathrin; heterotrimeric G-protein signaling pathway-Gi alpha and Gs alpha-mediated pathway→clathrin; Huntington disease→clathrin

P20674	COX5A_ HUMAN	Cytochrome C oxidase subunit 5A, mitochondrial	COX5A	Cytochrome C oxidase subunit 5A, mitochondrial (PTHR14200:SF11)	Oxidase(PC00176)	Oxidoreductase activity (GO:0003824)	Oxidative phosphorylation (GO:0008152); respiratory electron transport chain (GO:0006091)		

P07108	ACBP_ HUMAN	Acyl-CoA-binding protein	DBI	Acyl-CoA-binding protein (PTHR23310:SF54)	Transfer/carrier protein (PC00219); enzyme modulator (PC00095)	Catalytic activity (GO:0003824); protein binding (GO:0005488); enzyme inhibitor activity (GO:0005515)	Lipid metabolic process (GO:0008152); lipid transport (GO:0044238); regulation of catalytic activity (GO:0006629)		

Q15075	EEA1_ HUMAN	Early endosome antigen 1	EEA1	Early endosome antigen 1 (PTHR23164:SF6)					

Q92538	GBF1_ HUMAN	Golgi-specific brefeldin A-resistance guanine nucleotide exchange factor 1	GBF1	Golgi-specific brefeldin A-resistance guanine nucleotide exchange factor 1 (PTHR10663:SF138)	Signaling molecule (PC00207); guanyl-nucleotide exchange factor (PC00095); cytoskeletal protein (PC00022)	GTPase activity (GO:0003824); pyrophosphatase activity (GO:0016787); nucleotide binding (GO:0003924); small GTPase regulator activity (GO:0016462); guanyl-nucleotide exchange factor activity (GO:0005488)	Phosphate-containing compound metabolic process (GO:0008152); nitrogen compound metabolic process (GO:0006796); catabolic process (GO:0006807); nucleobase-containing compound metabolic process (GO:0009056); cellular process (GO:0044238); transport (GO:0006139); regulation of biological process (GO:0009987); regulation of catalytic activity (GO:0051179)	Organelle (GO:0043226); intracellular(GO:0044464)	

P78417	GSTO1_ HUMAN	Glutathione S-transferase omega-1	GSTO1	Glutathione S-transferase omega-1 (PTHR11260:SF323)	Transferase (PC00220); signaling molecule (PC00207); reductase (PC00176); translation elongation factor (PC00198); epimerase/ racemase (PC00171); cytoskeletal protein (PC00031)	Oxidoreductase activity (GO:0003824); transferase activity (GO:0016491); racemase and epimerase activity (GO:0016740); structural constituent of cytoskeleton (GO:0016853); translation elongation factor activity (GO:0016854); translation elongation factor activity (GO:0005198); receptor binding (GO:0005200); translation elongation factor activity (GO:0005488)	Immune system process (GO:0002376); translation (GO:0008152); cell communication (GO:0044238); response to toxic substance (GO:0019538); regulation of translation (GO:0006412)	Cytoskeleton (GO:0043226); intracellular (GO:0005856)	

P09211	GSTP1_ HUMAN	Glutathione S-transferase P	GSTP1	Glutathione S-transferase P (PTHR11571:SF141)					

P51858	HDGF_ HUMAN	Hepatoma-derived growth factor	HDGF	Hepatoma-derived growth factor (PTHR12550:SF41)	Transcription cofactor (PC00218); growth factor (PC00217)	Transcription cofactor activity (GO:0000988); sequence-specific DNA-binding transcription factor activity (GO:0000989); sequence-specific DNA-binding transcription factor activity (GO:0003712); growth factor activity (GO:0001071)	Immune system process (GO:0002376); transcription from RNA polymerase II promoter (GO:0008152); cell-cell signaling (GO:0044238); regulation of transcription from RNA polymerase II promoter (GO:0006139)		

O60814	H2B1K_ HUMAN	Histone H2B type 1-K	HIST1H2BK	Histone H2B type 1-K (PTHR23428:SF23)	Histone (PC00171)	Nucleic acid binding (GO:0005488)	Nitrogen compound metabolic process (GO:0008152); DNA metabolic process (GO:0006807); cellular process (GO:0044238); chromatin organization (GO:0006139); chromatin assembly (GO:0006259); cellular component biogenesis (GO:0009987)	Protein DNA complex (GO:0032991); nucleus (GO:0032993); nuclear chromosome (GO:0043226); intracellular (GO:0005634)	

P17096	HMGA1_ HUMAN	High mobility group protein HMG-I/HMG-Y	HMGA1	High mobility group protein HMG-I/HMG-Y (PTHR23341:SF1)	DNA-binding protein (PC00171)	Nucleic acid-binding transcription factor activity (GO:0001071); lyase activity (GO:0003824); DNA binding (GO:0016829)	Nitrogen compound metabolic process (GO:0008152); biosynthetic process (GO:0006807); DNA repair (GO:0009058); transcription, DNA-dependent (GO:0044238); cellular process (GO:0006139); response to stress (GO:0006259); regulation of nucleobase-containing compound metabolic process (GO:0006281)	Organelle (GO:0043226); intracellular (GO:0044464)	

P52926	HMGA2_ HUMAN	High mobility group protein HMGI-C	HMGA2	High mobility group protein HMGI-C (PTHR23341:SF4)	DNA-binding protein (PC00171)	Nucleic acid-binding transcription factor activity (GO:0001071); lyase activity (GO:0003824); DNA binding (GO:0016829)	Nitrogen compound metabolic process (GO:0008152); biosynthetic process (GO:0006807); DNA repair (GO:0009058); transcription, DNA-dependent (GO:0044238); cellular process (GO:0006139); response to stress (GO:0006259); regulation of nucleobase-containing compound metabolic process 2(GO:0006281)	Organelle (GO:0043226); intracellular (GO:0044464)	

P09429	HMGB1_ HUMAN	High mobility group protein B1	HMGB1	High mobility group protein B1-related (PTHR13711:SF164)	HMG box transcription factor (PC00218); signaling molecule (PC00024); chromatin/ chromatin-binding protein (PC00207)				p53 pathway→high mobility group protein 1

P05114	HMGN1_ HUMAN	Nonhistone chromosomal protein HMG-14	HMGN1	Nonhistone chromosomal protein HMG-14 (PTHR23087:SF12)	Chromatin/ chromatin- binding protein (PC00171)	Nucleic acid binding (GO:0005488); chromatin binding (GO:0003676)	DNA replication (GO:0008152); transcription from RNA polymerase II promoter (GO:0044238); cell cycle (GO:0006139)		

P05204	HMGN2_ HUMAN	Nonhistone chromosomal protein HMG-17	HMGN2	Nonhistone chromosomal protein HMG-17 (PTHR23087:SF13)	Chromatin/chromatin- binding protein (PC00171)	Nucleic acid binding (GO:0005488); chromatin binding (GO:0003676)	DNA replication (GO:0008152); transcription from RNA polymerase II promoter (GO:0044238); cell cycle (GO:0006139)		

P61604	CH10_ HUMAN	10 kDa heat shock protein, mitochondrial	HSPE1	10 kDa heat shock protein, mitochondrial (PTHR10772:SF16)	Chaperonin (PC00072)		Protein metabolic process (GO:0008152)		

P50213	IDH3A_ HUMAN	Isocitrate dehydrogenase [NAD] subunit alpha, mitochondrial	IDH3A	Isocitrate dehydrogenase [NAD] subunit alpha, mitochondrial (PTHR11835:SF34)	Dehydrogenase (PC00176)	Oxidoreductase activity (GO:0003824)	Generation of precursor metabolites and energy (GO:0008152); carbohydrate metabolic process (GO:0006091); tricarboxylic acid cycle (GO:0044238)		Ascorbate degradation→ L-xylulose-5-phosphate- 3-epimerase; leucine biosynthesis→ 3-isopropylmalate dehydrogenase

P01861	IGHG4_ HUMAN	Ig gamma-4 chain C region	IGHG4	IG gamma-1 chain c region-related (PTHR23266:SF83)					

Q9GZP8	IMUP_ HUMAN	Immortalization upregulated protein	IMUP	Immortalization upregulated protein (PTHR21830:SF0)					

P04732	MT1E_ HUMAN	Metallothionein-1E	MT1E	Metallothionein-1E (PTHR23299:SF2)					

P13640	MT1G_ HUMAN, MT1X_ HUMAN, MT2_ HUMAN	Metallothionein-1G, metallothionein-1X, metallothionein-2	MT1G	Metallothionein-1G (PTHR23299:SF3)					

P35749	MYH11_ HUMAN	Myosin-11	MYH11	Myosin-11 (PTHR13140:SF335)	G-protein modulator (PC00095); actin-binding motor protein (PC00022); cell junction protein (PC00085)	Motor activity (GO:0003824); structural constituent of cytoskeleton (GO:0016787); protein binding (GO:0016462); enzyme regulator activity (GO:0003774)	Metabolic process (GO:0008152); cytokinesis (GO:0009987); cellular component movement (GO:0000910); mitosis (GO:0006928); cell communication (GO:0007049); muscle contraction (GO:0007067); sensory perception of sound (GO:0007154); sensory perception (GO:0032501); mesoderm development (GO:0044707); anatomical structure morphogenesis (GO:0003008); muscle organ development (GO:0006936); intracellular protein transport (GO:0007605); vesicle-mediated transport (GO:0050877); regulation of catalytic activity (GO:0007600); cellular component organization (GO:0032502)	Plasma membrane (GO:0016020); actin cytoskeleton (GO:0005886); intracellular (GO:0043226)	Inflammation mediated by chemokine and cytokine signaling pathway→myosin; nicotinic acetylcholine receptor signaling pathway→myosin; cytoskeletal regulation by Rho GTPase→myosin light chain

P20929	NEBU_ HUMAN	Nebulin	NEB	Nebulin (PTHR11039:SF37)					

Q13442	HAP28_ HUMAN	28 kDa heat- and acid-stable phosphoprotein	PDAP1	28 kDa heat- and acid-stable phosphoprotein (PTHR22055:SF0)					

P13667	PDIA4_ HUMAN	Protein disulfide- isomerase A4	PDIA4	Protein disulfide-isomerase A4 (PTHR18929:SF110)		Isomerase activity (GO:0003824)	Protein folding (GO:0008152); cellular process (GO:0044238); response to stress (GO:0019538)	Organelle (GO:0043226); cytoplasm (GO:0044464)	

Q9UHV9	PFD2_ HUMAN	Prefoldin subunit 2	PFDN2	PREFOLDIN SUBUNIT 2 (PTHR13303:SF0)	Chaperone (PC00072)		protein folding (GO:0008152)		

O15212	PFD6_ HUMAN	Prefoldin subunit 6	PFDN6	Prefoldin subunit 6 (PTHR21431:SF0)		Protein binding (GO:0005488)	Protein complex assembly (GO:0008152); cellular process (GO:0044238); cellular component organization (GO:0019538); protein complex biogenesis (GO:0006461)	Protein complex (GO:0032991); cytosol (GO:0043234)	

Q01813	PFKAP_ HUMAN	ATP-dependent 6-phosphofructoki nase, platelet type	PFKP	6-Phosphofructokinase type C (PTHR13697:SF5)	Carbohydrate kinase (PC00220); carbohydrate kinase (PC00137)	Carbohydrate kinase activity (GO:0003824)	Glycolysis (GO:0008152); glycolysis (GO:0006091)		

P13797	PLST_ HUMAN	Plastin-3	PLS3	Plastin-3 (PTHR19961:SF32)	Nonmotor actin-binding protein (PC00085)	Structural constituent of cytoskeleton (GO:0005198); actin binding (GO:0005200)	Cellular process (GO:0009987); cellular component morphogenesis (GO:0032502); cellular component organization (GO:0009653)	Actin cytoskeleton (GO:0043226); intracellular (GO:0005856)	

O75334	LIPA2_ HUMAN	Liprin-alpha-2	PPFIA2	Liprin-alpha-2 (PTHR12587:SF6)			Cellular process (GO:0009987); cell adhesion (GO:0022610); ectoderm development (GO:0007155); nervous system development (GO:0032502)		

P30048	PRDX3_ HUMAN	Thioredoxin- dependent peroxide reductase, mitochondrial	PRDX3	Thioredoxin-dependent peroxide reductase, mitochondrial (PTHR10681:SF22)	Peroxidase (PC00176)	Oxidoreductase activity (GO:0003824); peroxidase activity (GO:0016491)	Metabolic process (GO:0008152)		

P49721	PSB2_ HUMAN	Proteasome subunit beta type-2	PSMB2	Proteasome subunit beta type-2 (PTHR11599:SF6)					
Q13200	PSMD2_ HUMAN	26S proteasome non-ATPase regulatory subunit 2	PSMD2	26S proteasome non-ATPase regulatory subunit 2 (PTHR10943:SF1)	Enzyme modulator (PC00095)	Catalytic activity (GO:0003824); protein binding (GO:0005488); enzyme regulator activity (GO:0005515)	Proteolysis (GO:0008152); cell cycle (GO:0044238); regulation of catalytic activity (GO:0019538)		Ubiquitin proteasome pathway→26S proteasome;

O00233	PSMD9_ HUMAN	26S proteasome non-ATPase regulatory subunit 9	PSMD9	26S proteasome non-ATPase regulatory subunit 9 (PTHR12651:SF1)	Enzyme modulator (PC00095)		Protein complex assembly (GO:0008152); cellular component organization (GO:0044238); protein complex biogenesis (GO:0019538)	Protein complex (GO:0032991); organelle (GO:0043234); intracellular (GO:0043226)	Ubiquitin proteasome pathway→26S proteasome;

P06454	PTMA_ HUMAN	Prothymosin alpha [cleaved into: prothymosin alpha, N-terminally processed; thymosin alpha-1]	PTMA	Prothymosin alpha (PTHR22745:SF0)			Nucleobase-containing compound metabolic process (GO:0008152)		

P20962	PTMS_ HUMAN	Parathymosin	PTMS	Parathymosin (PTHR22745:SF3)			Nucleobase-containing compound metabolic process (GO:0008152)		

P26373	RL13_ HUMAN	60S ribosomal protein L13	RPL13	60S ribosomal protein L13 (PTHR11722:SF0)	Ribosomal protein (PC00171)	Structural constituent of ribosome (GO:0005198); nucleic acid binding (GO:0003735)	Translation (GO:0008152)		

P62277	RS13_ HUMAN	40S ribosomal protein S13	RPS13	40S ribosomal protein S13 (PTHR11885:SF6)	Ribosomal protein (PC00171)	Structural constituent of ribosome (GO:0005198); nucleic acid binding (GO:0003735)	Protein metabolic process (GO:0008152)		

P62857	RS28_ HUMAN	40S ribosomal protein S28	RPS28	40S ribosomal protein S28 (PTHR10769:SF4)	Ribosomal protein (PC00171)	Structural molecule activity (GO:0005198)	Cellular process (GO:0009987); RNA localization (GO:0051179); nucleobase-containing compound transport (GO:0006403); nuclear transport (GO:0006810); cellular component biogenesis (GO:0015931)	Ribosome (GO:0032991); organelle (GO:0030529); cytosol (GO:0005840)	

Q8NC51	PAIRB_ HUMAN	Plasminogen activator inhibitor 1 RNA-binding protein	SERBP1	Plasminogen activator inhibitor 1 RNA-binding protein (PTHR12299:SF29)	RNA-binding protein (PC00171)	RNA binding (GO:0005488)	Primary metabolic process (GO:0008152)		

Q9H299	SH3L3_ HUMAN	SH3 domain- binding glutamic acid-rich-like protein 3	SH3BGRL3	SH3 domain-binding glutamic acid-rich-like protein 3 (PTHR12232:SF3)					

P11166	GTR1_ HUMAN	Solute carrier family 2, facilitated glucose transporter member 1	SLC2A1	Solute carrier family 2, facilitated glucose transporter member 1 (PTHR23503:SF51)					Gonadotropin releasing hormone receptor pathway→Glut1; gonadotropin releasing hormone receptor pathway→Glut1; gonadotropin releasing hormone receptor pathway→Glut1

P22528	SPR1B_ HUMAN	Cornifin-B	SPRR1B	Cornifin-B (PTHR23263:SF47)					

Q9Y6N5	SQRD_ HUMAN	Sulfide:quinone oxidoreductase, mitochondrial	SQRDL	Sulfide:quinone oxidoreductase, mitochondrial (PTHR10632:SF2)					

Q16629	SRSF7_ HUMAN	Serine/arginine- rich splicing factor 7	SRSF7						

P16949	STMN1_ HUMAN	Stathmin	STMN1	Stathmin (PTHR10104:SF5)		Cytoskeletal protein binding (GO:0005488)	Cellular process (GO:0009987); single-multicellular organism process (GO:0032501); cell differentiation (GO:0044707); nervous system development (GO:0032502); regulation of biological process (GO:0030154); cytoskeleton organization (GO:0048731)	Intracellular (GO:0044464); cell projection (GO:0005622)	Cytoskeletal regulation by Rho GTPase→stathmin

P61956	SUMO2_ HUMAN	Small ubiquitin-related modifier 2	SUMO2	Small ubiquitin-related modifier 2-related (PTHR10562:SF11)			Cellular protein modification process (GO:0008152); cell cycle (GO:0044238)		

P63313	TYB10_ HUMAN	Thymosin beta-10	TMSB10	Thymosin beta-10 (PTHR12021:SF10)					

P62328	TYB4_ HUMAN	Thymosin beta-4	TMSB4X	Thymosin beta-4 (PTHR12021:SF13)					

P67936	TPM4_ HUMAN	Tropomyosin alpha-4 chain	TPM4	Tropomyosin alpha-4 chain (PTHR19269:SF40)	Actin-binding motor protein (PC00085)	Motor activity (GO:0003824); structural constituent of cytoskeleton (GO:0016787)	Metabolic process (GO:0008152); cellular component movement (GO:0009987); muscle contraction (GO:0006928); ectoderm development (GO:0032501); mesoderm development (GO:0044707); cellular component morphogenesis (GO:0003008); nervous system development (GO:0006936); muscle organ development (GO:0032502); cellular component organization (GO:0007398)	Actin cytoskeleton (GO:0043226); intracellular (GO:0005856)	
Q9C030	TRIM6_ HUMAN	Tripartite motif-containing protein 6	TRIM6	Tripartite motif-containing protein 6 (PTHR24103:SF348)					

Q13509	TBB3_ HUMAN	Tubulin beta-3 chain	TUBB3	Tubulin beta-3 chain (PTHR11588:SF43)	Tubulin (PC00085)	Structural constituent of cytoskeleton (GO:0005198)	Cell cycle (GO:0009987); anatomical structure morphogenesis (GO:0007049); intracellular protein transport (GO:0032502)	Protein complex (GO:0032991); intracellular (GO:0043234)	Huntington disease→beta-tubulin; Huntington disease→microtubule; cytoskeletal regulation by Rho GTPase→tubulin

O75152	ZC11A_ HUMAN	Zinc finger CCCH domain- containing protein 11A	ZC3H11A	Zinc finger CCCH domain-containing protein 11A (PTHR15725:SF2)	Nucleic acid binding (PC00171)	Nucleic acid binding (GO:0005488)			

**Table 4:** List of top enriched pathways provided after overrepresentation analysis with PathVisio. Positive (*r*) is the number of genes in the pathway meeting the criterion. Measured (*n*) is the number of genes in the pathway measured in the experiment. Total is the total number of elements in the pathway. *Z*-score is the score calculated for overrepresentation analysis. Pathways with a high *Z*-score have more significantly up- or downregulated genes than expected.

Pathway	Positive (*r*)	Measured (*n*)	Total	%	*Z*-score	*p* value (permuted)
Zinc homeostasis	4	4	39	100.00%	3.30	0.000
Copper homeostasis	5	6	58	83.33%	3.13	0.000
Arachidonate epoxygenase/epoxide hydrolase	2	2	17	100.00%	2.33	0.001
DNA replication	2	2	50	100.00%	2.33	0.009
Retinoblastoma (RB) in cancer	2	2	98	100.00%	2.33	0.012
Histone modifications	11	25	69	44.00%	1.98	0.027
Aryl hydrocarbon receptor	1	1	51	100.00%	1.65	0.048
Cardiac hypertrophic response	1	1	60	100.00%	1.65	0.063
Constitutive androstane receptor pathway	1	1	34	100.00%	1.65	0.029
Dual hijack model of Vif in HIV infection	1	1	9	100.00%	1.65	0.007
Endochondral ossification	1	1	69	100.00%	1.65	0.072
Endothelin pathways	1	1	47	100.00%	1.65	0.047
Gastric cancer network 1	1	1	32	100.00%	1.65	0.027
Melatonin metabolism and effects	1	1	55	100.00%	1.65	0.057
NOTCH1 regulation of human endothelial cell calcification	1	1	18	100.00%	1.65	0.014
Notch signaling pathway	1	1	62	100.00%	1.65	0.058
RalA downstream regulated genes	1	1	13	100.00%	1.65	0.015
T-cell receptor and costimulatory signaling	1	1	45	100.00%	1.65	0.050
TarBasePathway	1	1	19	100.00%	1.65	0.019
Type II interferon signaling (IFNG)	1	1	38	100.00%	1.65	0.043
Metapathway biotransformation	2	3	189	66.67%	1.55	0.120
Cytoplasmic ribosomal proteins	13	34	89	38.24%	1.54	0.121
Circadian rythm-related genes	5	11	210	45.45%	1.40	0.146
Apoptosis modulation and signaling	3	6	97	50.00%	1.28	0.190
Alzheimers disease	4	9	163	44.44%	1.19	0.259
Electron transport chain	4	9	118	44.44%	1.19	0.252
Oxidative stress	2	4	32	50.00%	1.04	0.232
Preimplantation embryo	2	4	60	50.00%	1.04	0.299
Vitamin B12 metabolism	2	4	118	50.00%	1.04	0.337
TNF alpha signaling pathway	3	7	97	42.86%	0.95	0.340
